# The Effects of Genetic Mutations and Drugs on the Activity of the Thiamine Transporter, SLC19A2

**DOI:** 10.1208/s12248-021-00562-4

**Published:** 2021-03-01

**Authors:** Osatohanmwen J. Enogieru, Megan L. Koleske, Bianca Vora, Huy Ngo, Sook Wah Yee, Derrick Chatad, Marina Sirota, Kathleen M. Giacomini

**Affiliations:** 1 Department of Bioengineering and Therapeutic Sciences, University of California at San Francisco, San Francisco, California 94158 USA; 2 College of Medicine, SUNY Downstate Health Sciences University, Brooklyn, New York 11203 USA; 3 Department of Pediatrics, University of California at San Francisco, San Francisco, California 94158 USA; 4 Institute of Human Genetics, University of California at San Francisco, San Francisco, California 94158 USA; 5 Bakar Computational Health Sciences Institute, University of California at San Francisco, San Francisco, California, 94158 USA

**Keywords:** drug nutrient interactions, drug-induced megaloblastic anemia, thiamine diphosphate, THTR1, vitamin b1

## Abstract

A rare cause of megaloblastic anemia (MA) is thiamine-responsive megaloblastic anemia (TRMA), a genetic disorder caused by mutations in *SLC19A2* (encoding THTR1), a thiamine transporter. The study objectives were to (1) functionally characterize selected TRMA-associated SLC19A2 variants and (2) determine whether current prescription drugs associated with drug-induced MA (DIMA) may act via inhibition of SLC19A2. Functional characterization of selected SLC19A2 variants was performed by confocal microscopy and isotopic uptake studies of [3H]-thiamine in HEK293 cells. Sixty-three drugs associated with DIMA were screened for SLC19A2 inhibition in isotopic uptake studies. Three previously uncharacterized SLC19A2 variants identified in TRMA patients exhibited disrupted localization to the plasma membrane along with near-complete loss-of-function. Ten of 63 drugs inhibited SLC19A2-mediated thiamine transport ≥ 50% at screening concentrations; however, with the exception of erythromycin, none was predicted to inhibit SLC19A2 at clinically relevant unbound plasma concentrations. Data from electronic health records revealed reduced levels of thiamine pyrophosphate (TPP) in patients prescribed erythromycin, consistent with inhibition of SLC19A2-mediated thiamine transport. Here, we confirmed the role of three SLC19A2 variants in TRMA pathology. Additionally, we report that inhibition of SLC19A2 is a potential, but uncommon mechanism for DIMA.

**Supplementary Information:**

The online version contains supplementary material available at 10.1208/s12248-021-00562-4.

## INTRODUCTION

**Fig. 1:**
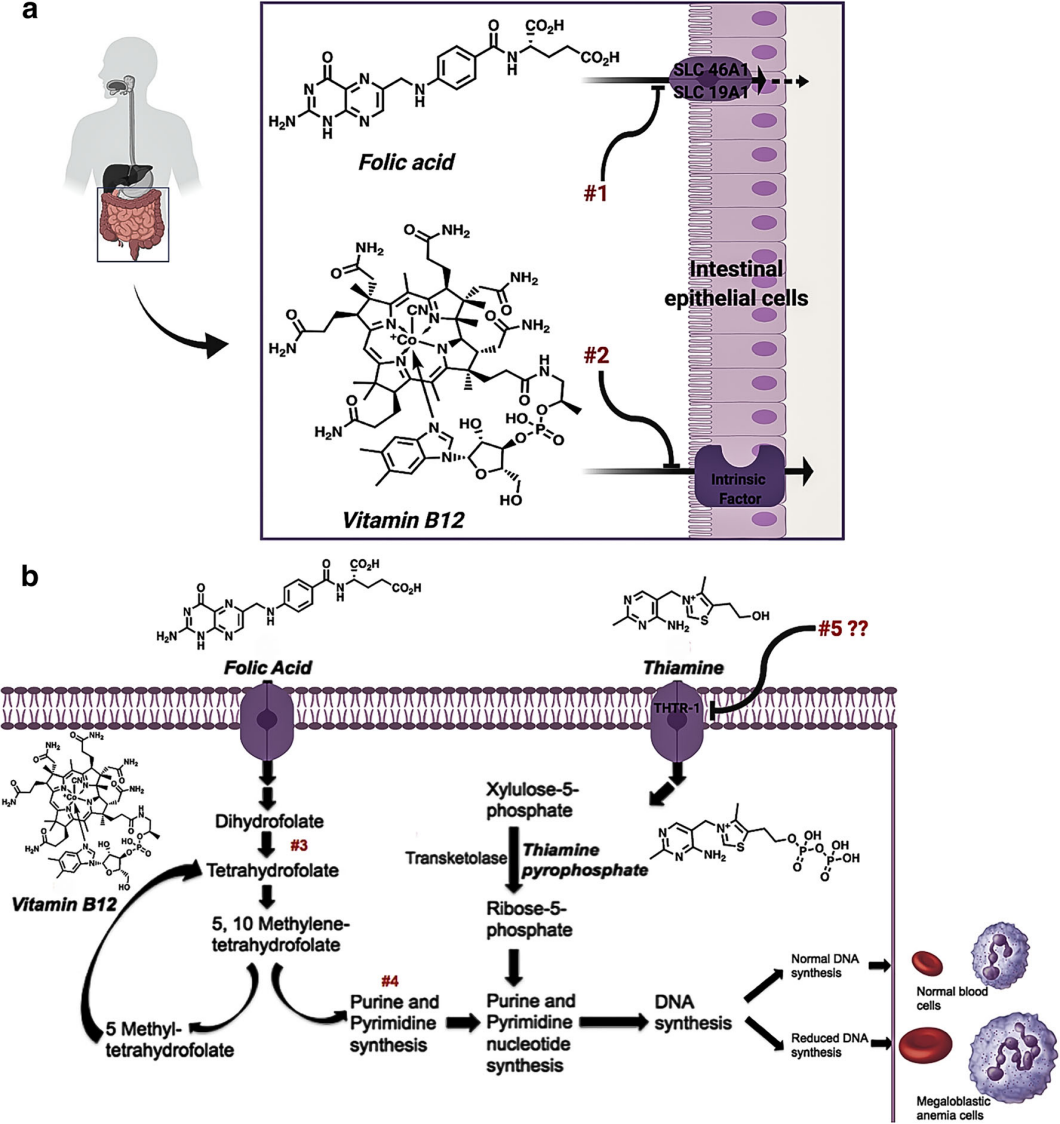
Common mechanisms underlying megaloblastic anemia and drug-induced megaloblastic anemia. Major causes of megaloblastic anemia (MA) include deficiencies of vitamin B_12_ and folic acid due to inadequate dietary intake or malabsorption. MA due to drug exposure is also known as drug-induced megaloblastic anemia (DIMA). DIMA can be caused by a variety of drugs that inhibit DNA synthesis at different points of the DNA synthesis pathway. **a** Drugs can cause MA by reducing cellular availability of vitamin B_12_ or folic acid through reduced absorption, plasma transport, or delivery of folate or vitamin B_12_ or physical destruction of the vitamins (mechanisms #1 and #2). **b** Additional mechanisms of DIMA include inhibition of key enzymes such as dihydrofolate reductase resulting in tetrahydrofolate deficiency (mechanism #3) and drugs acting as purine and pyrimidine antagonists or analogs, including chemotherapies (5-fluorouracil), immune antagonists (e.g., leflunomide), and antiviral agents (mechanism #4). Interestingly, some drugs cause MA through an unknown mechanism. We propose an unknown mechanism of DIMA may involve inhibition of SLC19A2 in hematopoietic cells (mechanism #5)

Megaloblastic anemia (MA) describes a heterogeneous set of anemias characterized by the presence of megaloblasts (immature red blood cells) in the bone marrow. MA develops as a result of ineffective DNA synthesis in rapidly dividing hematopoietic cells (1). Major causes of MA include vitamin deficiencies and exposure to certain drugs (2), termed drug-induced megaloblastic anemia (DIMA). As nutritional deficiencies have decreased in Western populations, the percentage of MA caused by drugs (i.e., DIMA) has increased (2, 3). DIMA occurs through various mechanisms, some of which remain elusive (Fig. 1).

Thiamine deficiency can be caused by poor dietary intake, chronic alcoholism, poor absorption (e.g., gastric bypass surgery or diarrhea), increased utilization (e.g., pregnancy), genetic mutations, and use of particular medications (e.g., diuretics) (4). Thiamine deficiency usually occurs systemically but it can occur locally in specific tissue/organs. Systemic thiamine deficiency can cause disorders such as beriberi and Wernicke-Korsakoff syndrome, whereas tissue/organ-specific thiamine deficiency due to genetic inactivation of thiamine transporters can lead to thiamine metabolism dysfunction syndrome-2 (THMD2, OMIM# 607483) or thiamine-responsive megaloblastic anemia (TRMA, OMIM# 249270) (4).

TRMA is characterized by three hallmark signs: MA, non-autoimmune type 1 diabetes, and sensorineural hearing loss (5). TRMA is caused by loss-of-function genetic mutations in *SLC19A2*, encoding the thiamine human transporter 1 (THTR1, SLC19A2) (6). SLC19A2 is the main thiamine transporter in pancreatic islet tissue and hematopoietic cells (7, 8). Approximately 60 *SLC19A2* variants have been associated with TRMA (Supplemental Table 1) (5, 9), yet few have been functionally characterized to date. A mouse model of dietary thiamine deficiency (TD) has been used extensively to evaluate the effects of systemic thiamine depletion on neurological functions (10). However, there are no studies employing the TD model (or any *in vivo* system) to evaluate the impact of systemic thiamine depletion on erythropoiesis and megaloblastic anemia. Further, no studies have explored the idea that pharmacologic inhibitors of thiamine transporters may be causal for megaloblastic anemia (4). To our knowledge, SLC19A2 inhibition, which may phenocopy loss-of-function *SLC19A2* variants, is not a known mechanism for DIMA.

In this study, we tested the hypothesis that drugs associated with DIMA may act (at least in part) as inhibitors of SLC19A2, and phenocopy genetic variants in the transporter. The objectives of our study were to (1) functionally characterize selected *SLC19A2* variants associated with TRMA and (2) identify inhibitors of SLC19A2 among drugs associated with DIMA. The current study extends our work on thiamine transport mechanisms with a focus on THTR1 (SLC19A2). In particular, we functionally characterize several genetic mutations in *SLC19A2* and identify clinically relevant SLC19A2 inhibitors. Collectively, our studies expand our understanding of drug-induced thiamine disorders.

## MATERIALS AND METHODS

### Chemicals and Reagents

[^3^H]-Thiamine hydrochloride (catalog #ART 0710) was purchased from American Radiolabeled Chemicals Incorporation (St. Louis, MO, USA). Non-radiolabeled compounds were purchased from Sigma-Aldrich, Inc. (USA), Selleck Chemicals (Houston, TX), VWR International, Inc., and Thermo Fisher Scientific (USA). Cell culture supplies were purchased from Thermo Fisher Scientific (USA).

### Functional Studies

Plasmids encoding wild-type *SLC19A2* and three TRMA-associated variants were generated by Genscript Inc. (Piscataway, USA). Methods for generation of stably overexpressing SLC19A2 cell lines and uptake studies have been previously described (11, 12). Inhibition and kinetic studies were performed in SLC19A2-overexpressing stable HEK293 cells transiently transfected with an additional 500 ng of *SLC19A2* wild-type plasmid.

### Confocal Microscopy of SLC19A2 GFP-Tagged Variants

The subcellular localization of wild-type and variant SLC19A2 was determined with confocal microscopy on a Nikon CSU-22 Spinning Disk. Samples were prepared as previously described (12).

### Inhibitor Screening Studies

Prescription drugs associated with MA (2) in addition to others belonging to listed drug classes (e.g., proton-pump inhibitors; 63 compounds total) were screened in triplicate at 1 mM to evaluate inhibitory activity against SLC19A2. The screen was performed using [^3^H]-thiamine (25 nM). Each plate contained control wells exposed for 10 min (within linear range, Supplemental Figure 1) to [^3^H]-thiamine (positive control), [^3^H]-thiamine plus 1 mM of amprolium (negative control), and [^3^H]-thiamine plus 1 mM of screened drug (3 replicates per drug). Methods have been previously described (11). SLC19A2 inhibitors were defined as compounds that decreased SLC19A2*-*mediated thiamine uptake by 50% or more at 1 mM. For compounds that met this definition, inhibition assays were conducted and IC_50_ values were calculated by nonlinear fitting using GraphPad Prism 7 (La Jolla, CA) (11).

### Calculation of SLC19A2 Inhibitor Systemic Blood Concentrations

Clinical pharmacokinetic data from human studies (13–18) and drug monograph databases (e.g., Micromedex, FDA) were used to obtain *C*_max_ (maximum plasma concentration) and *f*_u_ (fraction of drug unbound in the plasma). Comparisons between unbound *C*_max_ (*f*_u_*C_max_) and IC_50_ obtained *in vitro* ((*f*_u_*C_max_)/IC_50_ > 0.1) were used to estimate the likelihood that a drug may inhibit SLC19A2 clinically (Table I) (19, 20).

**Table I:** Predicted Risk of Causing an *SLC19A2*-Mediated Clinical Drug-Drug Interaction Using the FDA Criteria (19, 20)

Drug	*f*_u_	*C*_max_, μM	IC_50_, μM	*(*f*_u_ * *C*_max_) ÷ (IC_50_)	Reference
Erythromycin	0.1-0.3	2.3-9.6	20	0.144	PMID: 11294369 PMID: 3606934 PMID: 2656049
Mycophenolate mofetil	0.03 (label)	Below LLQ (0.9 μM)	145	NA	Package insert
Omeprazole	0.05 (label)	1-10	207	0.002	PMID: 1458764 PMID: 8675169
Chloroquine	0.36-0.39	0.0034	301	4*10^-6^	PMID: 23701202 PMID: 6849768
Pantoprazole	0.02 (label)	6.5	429	0.0003	Package insert
Amiloride	0.77	0.04	69	0.0004	Package insert
Trimethoprim	0.56 (label)	3.44	6.84	0.28	PMID: 27803021 Package insert

FDA criteria* (19, 20) adapted for use in predicting the potential of a drug to cause a transporter-mediated drug-vitamin interaction suggest that if the value in this column is > 0.1, the drug has the potential to cause a clinical drug-vitamin interaction

*NA* not applicable, *LLQ* lower limit of quantification

### EHR Data Analyses

Methods detailing electronic health record (EHR) data extraction, filtering, and analysis are listed in Supplemental Table 2 and Supplemental Table 3.

### TPK1 Enzyme Assay

Methods detailing thiamine pyrophosphokinase 1 (TPK1) enzyme assay development and execution are listed in Supplemental Figure 2 legend.

## RESULTS

### *SLC19A2* Variants Causal for TRMA Exhibit Complete Loss-of-Function

**Fig. 2:**
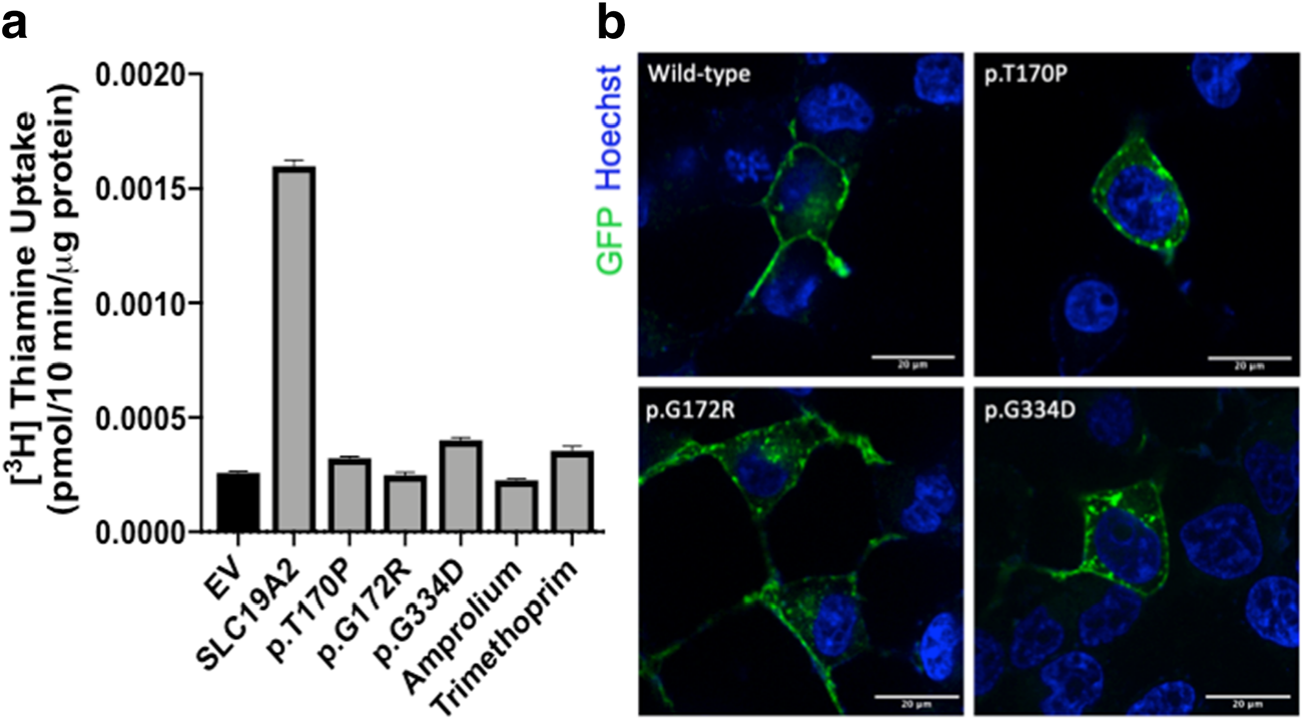
Function and expression of TRMA-associated *SLC19A2* variants, and the effects of two prescription drugs on SLC19A2-mediated thiamine uptake. HEK293 cells transiently transfected with TRMA-associated *SLC19A2* variants (p.T170P, p.G172R, and p.G334D) had significantly reduced thiamine transport compared to cells transfected with wild-type *SLC19A2* transporter (one-way ANOVA *p*-value < 0.0001); functions of SLC19A2 variants were similar to that of empty vector cells (Student’s *t*-test EV *versus* p.G172R, *p*-value = 0.49). At 200 μM, the known SLC19A2 inhibitors trimethoprim and amprolium abolished thiamine transport by wild-type SLC19A2 resulting in transporter function comparable to the TRMA variants. Figures representative of 3 separate experiments (**a**). Membrane localization of GFP-tagged SLC19A2 variants. To determine TRMA variant membrane expression, constructs encoding *SLC19A2* wild-type and TRMA variants were GFP-tagged and transfected in HEK293 cells. Nuclei were stained with Hoechst 33342 dye (blue) and cells were visualized with confocal microscopy (**b**)

HEK293 cells transiently transfected with three previously uncharacterized TRMA-associated *SLC19A2* variants (p.T170P, p.G172R, and p.G334D) had significantly reduced thiamine transport activity compared to cells transfected with wild-type *SLC19A2* (one-way ANOVA *p*-value < 0.0001, Fig. 2). The activities of variants p.T170P and p.G172R were not significantly different from cells transfected with empty vector (Fig. 2a; Student’s *t*-test *p* = 0.052 and 0.49, respectively), while variant p.G334D retained minimal but significant thiamine transport (*p* =0.0009). Similarly, inhibition of SLC19A2 with 200 μM amprolium or trimethoprim (a commonly used antibiotic and SLC19A2 inhibitor) abolished wild-type SLC19A2 activity to levels comparable to the TRMA-associated variants (Fig. 2a).

### *SLC19A2* Variants Exhibit Disrupted Plasma Membrane Localization

Confocal microscopy of HEK293 cells expressing GFP-tagged wild-type and variant SLC19A2 revealed that the wild-type SLC19A2 localized primarily to the plasma membrane of the cell (Fig. 2b). In contrast, all three SLC19A2 variants exhibited partially disrupted membrane localization, and displayed concentrated puncta consistent with retention of the transporter variants in membrane-bound organelles.

### Inhibitor Screen Identifies Ten Drugs That Substantially Inhibit *SLC19A2*

**Fig. 3:**
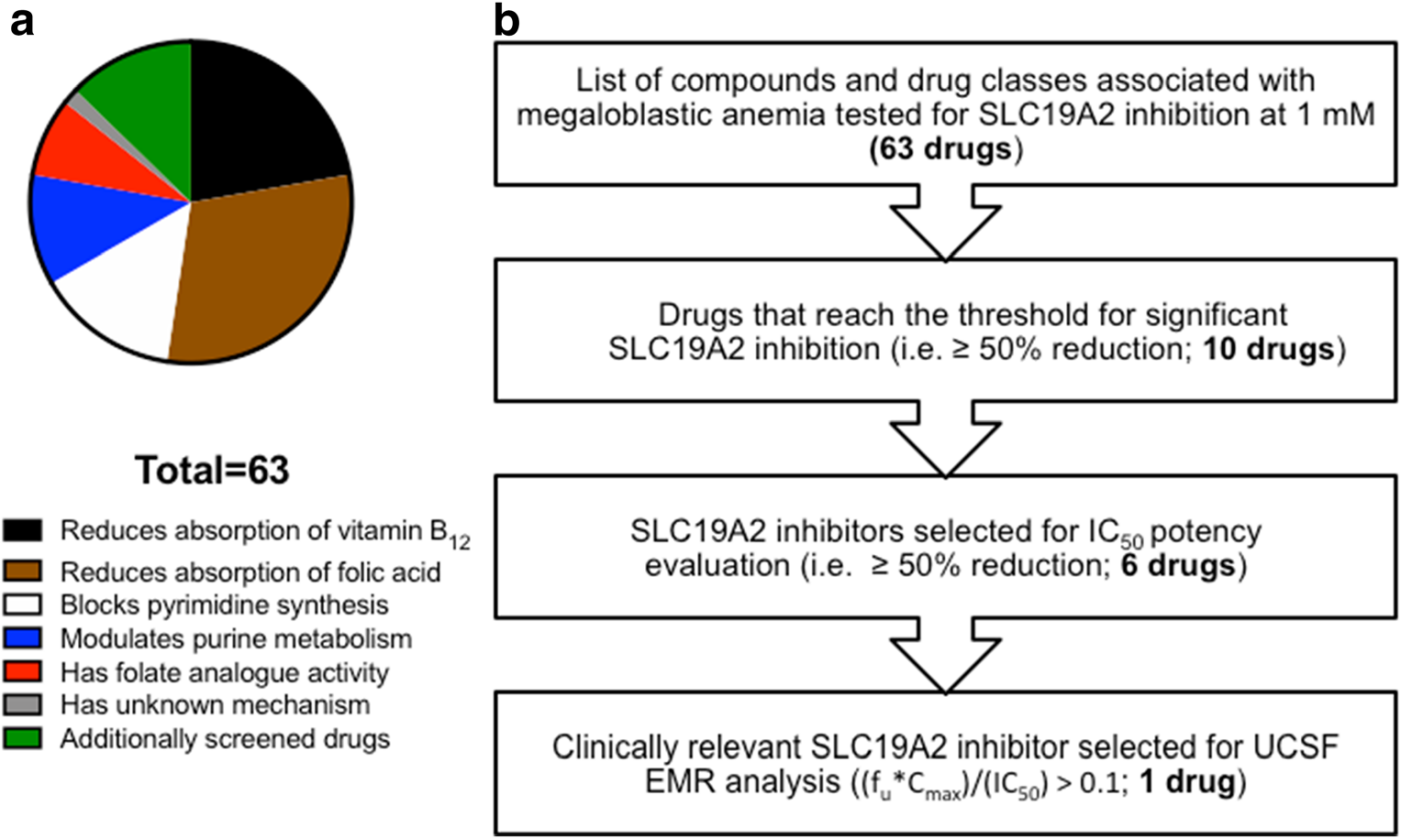
Common mechanisms of drug-induced megaloblastic anemia and SLC19A2 inhibitor drug screen workflow. A list of drugs associated with inducing megaloblastic anemia through various mechanisms was compiled (**a**). Each drug was dissolved to a final concentration of 1 mM in buffer containing radiolabeled [^3^H] thiamine and exposed to HEK293 cells expressing SLC19A2 wild-type transporter for 10 min. Drugs that inhibited 50% or more of SLC19A2-mediated thiamine uptake were considered significant inhibitors (10 total). Next, IC_50_ experiments were conducted for the top six inhibitors in the screen. Last, we selected SLC19A2 inhibitors for EHR analysis that reached the following threshold: (*f*_u_*C_max_)/(IC_50_) > 0.1 (**b**)

**Fig. 4:**
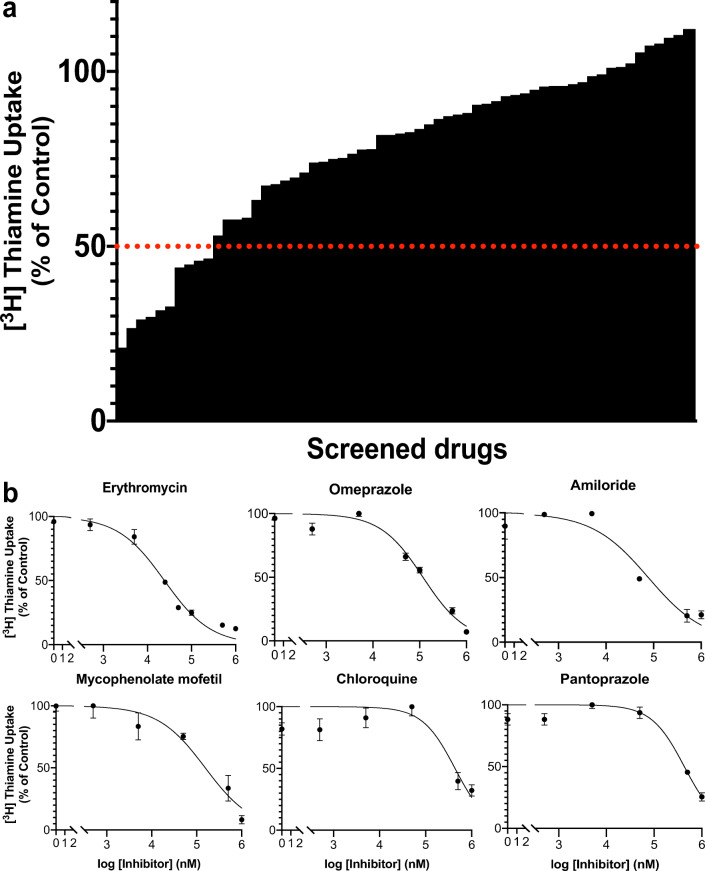
Drug screen and potency studies against SLC19A2-mediated thiamine uptake. In the screen, SLC19A2 inhibitors were defined as compounds that inhibit at least 50% of thiamine uptake compared to the positive control. The positive control was thiamine uptake in the absence of inhibitor. Out of the 63 drugs screened, ten reached that threshold. Of the ten drugs, three (fedratinib, amprolium, and trimethoprim) have IC_50_ values published in literature (**a**). IC_50_ experiments were conducted using inhibitor concentrations from 0 to 1 mM. IC_50_ curves for erythromycin, omeprazole, amiloride, mycophenolate mofetil, chloroquine, and pantoprazole were generated by fitting data to a nonlinear model (**b**). Figures representative of 3 independent experiments except for pantoprazole

Among the 63 drugs screened for SLC19A2 inhibition (Fig. 3, Fig. 4, Supplemental Table 4), ten were designated as inhibitors, that is, these 10 substantially inhibited SLC19A2 (≥ 50% reduction in thiamine transport) at 1 mM. Of the ten inhibitors, four (fedratinib, amiloride, trimethoprim, amprolium) were previously identified as SLC19A2 inhibitors (11, 20), whereas six were novel SLC19A2 inhibitors, including erythromycin, mycophenolate mofetil, omeprazole, pantoprazole, lansoprazole, and chloroquine.

### IC_50_ Assays Reveal Erythromycin Potently Inhibits SLC19A2

Out of all the newly identified SLC19A2 inhibitors, erythromycin was the most potent with an estimated IC_50_ of 20 μM. Inhibition potency was followed by amiloride (69 μM), mycophenolate mofetil (145 μM), omeprazole (207 μM), chloroquine (301 μM), and pantoprazole (429 μM) (Table I).

### Erythromycin Reaches Systemic Concentrations Relevant for SLC19A2 Inhibition

Calculation of unbound *C*_max_/IC_50_ values for the SLC19A2 inhibitors listed in Table I revealed that erythromycin was the only drug that met the FDA’s criteria for clinically relevant transporter inhibition.

### Erythromycin Is a Substrate of TPK1

Luminescent signals from each enzyme reaction were measured and compared for omeprazole, erythromycin, thiamine (positive control or PC), and no substrate (negative control or NC). Luminescent signal in the erythromycin reaction was significantly higher than the negative control signal (Supplemental Figure 2, Student’s *t*-test, *p*-value < 0.0001), but still lower than the signal from the positive control (thiamine 1 mM). For omeprazole, the signal was statistically similar to the negative control (Supplemental Figure 2, Student’s *t*-test, *p*-value = 0.72).

## DISCUSSION

In our previous research, we discovered that thiamine is the major endogenous substrate of OCT1 transporters (21), identified OCT1 as a key determinant of certain cardiometabolic (22), and hepatic (23) traits and identified several prescription drugs that are clinically relevant drug inhibitors of the intestinal thiamine transporter, SLC19A3 (24). In this study, we expand on our previous research by evaluating SLC19A2 expression and function and the physiological effects of SLC19A2 inhibition.

Approximately half of the 60 *SLC19A2* mutations associated with TRMA are missense variants (Supplemental Table 1) (5, 25), yet few have been functionally characterized. In this study, we determined that three previously uncharacterized TRMA-associated *SLC19A2* missense variants exhibited near-complete loss-of-function (Fig. 2). Consistent with loss-of-function, confocal microscopy revealed that the *SLC19A2* variants largely failed to traffic to the plasma membrane. Instead, intracellular puncta were observed for all three variants, suggestive of impaired transporter trafficking or recycling, a pattern that has been observed previously for mutations in *SLC19A2* (26, 27).

**Fig. 5:**
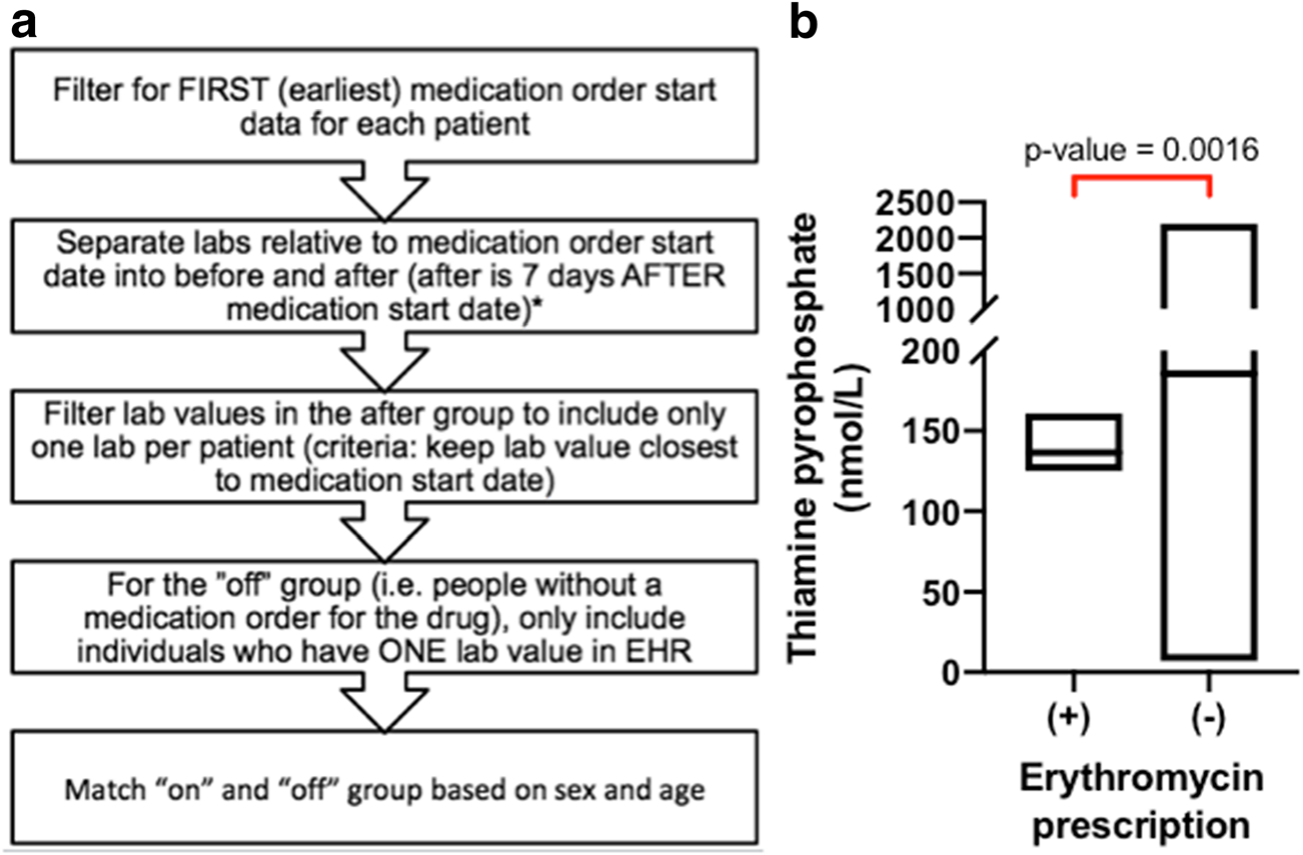
Methods used to extract information from electronic health records (EHR) and results of thiamine pyrophosphate levels in patients prescribed erythromycin. To explore whether drugs predicted to cause a clinical drug-drug interaction in *in vitro* studies inhibit SLC19A2 *in vivo*, we extracted and analyzed data from the UCSF EHR. Data was filtered by presence (“on”) or absence (“off”) of erythromycin prescription, medication order start date, and thiamine pyrophosphate laboratory data. Before analysis, individuals in the “on” and “off” group were age and sex-matched (**a**). The thiamine pyrophosphate levels of patients prescribed erythromycin (+, *N* = 4) *versus* not prescribed erythromycin (−, *N* = 200) were compared. The average TPP levels for patients prescribed erythromycin were lower (137 nM) than that of patients not prescribed erythromycin (186 nM) (Welch’s two sample *t*-test, *p*-value = 0.0016)

In the inhibitor screen, three prescription drugs associated with DIMA had IC_50_ values of < 200 μM. However, only the macrolide antibiotic, erythromycin, was predicted to inhibit SLC19A2 at clinically relevant concentrations when applying the FDA criteria for transporter-mediated drug-drug interactions. Erythromycin-induced MA is thought to occur through inhibition of folic acid absorption (2). However, our data suggest an additional mechanism via SLC19A2 inhibition (Fig. 4b), and potentially from inhibition of TPK1 (the enzyme that converts thiamine to TPP, the bioactive form of thiamine) (Supplemental Figure 2). Our finding that erythromycin is a substrate of TPK1 (Supplemental Figure 2) together with the fact that the drug is known to accumulate intracellularly to high levels (28) suggests that inhibition of TPK1 as well as of SLC19A2 may contribute to low TPP levels and ultimately to erythromycin-induced MA. EHR data were consistent with reduced levels of TPP in patients treated with erythromycin (Fig. 5, Supplemental Table 2, Supplemental Table 3). This mechanism may be particularly important in patients who may have higher systemic levels of erythromycin (e.g., patients with liver disease, the elderly, CYP3A4 poor metabolizers). Today, erythromycin use is rare compared to other macrolides (e.g., azithromycin).

## CONCLUSION

Three TRMA-associated *SLC19A2* variants were characterized, revealing complete loss of thiamine transport at least partially attributed to disrupted membrane localization. Our data suggest that SLC19A2 inhibition is not the mechanism behind DIMA for most associated prescription drugs, although it may contribute to erythromycin-induced MA.

## Supplementary Information

ESM 1 (DOC 290 kb)

## References

[1] Green R. Megaloblastic anemias nutritional and other causes. Med Clin NA [Internet]. 2017;101(2):297–317. Available from: 10.1016/j.mcna.2016.09.013, 2017 10.1016/j.mcna.2016.09.013 28189172

[2] Hesdorffer CS, Longo DL (2015). Drug-induced megaloblastic anemia. N Engl J Med.

[3] Ayed S, Qahtani A (2018). Drug-induced megaloblastic, aplastic, and hemolytic anemias: current concepts of pathophysiology and treatment. Int J Clin Exp Med.

[4] Wiley KD, Gupta M. Vitamin B1 thiamine deficiency. In Treasure Island (FL); 2020.

[5] Raz T, Labay V, Baron D, Szargel R, Anbinder Y, Barrett T, Rabl W, Viana MB, Mandel H, Baruchel A, Cayuela JM, Cohen N (2000). The spectrum of mutations, including four novel ones, in the Thiamine-responsive megaloblastic anemia gene SLC19A2 of eight families. Hum Mutat.

[6] Labay V, Raz T, Baron D, Mandel H, Williams H, Barrett T, Szargel R, McDonald L, Shalata A, Nosaka K, Gregory S, Cohen N (1999). Mutations in SLC19A2 cause thiamine-responsive megaloblastic anaemia associated with diabetes mellitus and deafness. Nat Genet.

[7] Mee L, Nabokina SM, Sekar VT, Subramanian VS, Maedler K, Said HM (2009). Pancreatic beta cells and islets take up thiamin by a regulated carrier-mediated process: studies using mice and human pancreatic preparations. Am J Physiol Gastrointest Liver Physiol.

[8] Uhlen M, Fagerberg L, Hallstrom BM, Lindskog C, Oksvold P, Mardinoglu A, et al. Proteomics. Tissue-based map of the human proteome. Science [Internet]. 2015 Jan;347(6220):1260419. Available from: https://www.proteinatlas.org/ENSG00000117479-SLC19A2/blood 10.1126/science.1260419 25613900

[9] Marcé-Grau A, Martí-Sánchez L, Baide-Mairena H, Ortigoza-Escobar JD, Pérez-Dueñas B (2019). Genetic defects of thiamine transport and metabolism: a review of clinical phenotypes, genetics, and functional studies. J Inherit Metab Dis.

[10] Moraes JO, Rodrigues SDC, Pereira LM, Medeiros R (2018). de CN, de Cordova CAS, de Cordova FM. Amprolium exposure alters mice behavior and metabolism in vivo. Anim Model Exp Med.

[11] Liang X, Chien H-C, Yee SW, Giacomini MM, Chen EC, Piao M, Hao J, Twelves J, Lepist EI, Ray AS, Giacomini KM (2015). Metformin is a substrate and inhibitor of the human thiamine transporter, THTR-2 (SLC19A3). Mol Pharm.

[12] Urban TJ, Gallagher RC, Brown C, Castro RA, Lagpacan LL, Brett CM, Taylor TR, Carlson EJ, Ferrin TE, Burchard EG, Packman S, Giacomini KM (2006). Functional genetic diversity in the high-affinity carnitine transporter OCTN2 (SLC22A5). Mol Pharmacol.

[13] Landahl S, Andersson T, Larsson M, Lernfeldt B, Lundborg P, Regardh CG (1992). Pharmacokinetic study of omeprazole in elderly healthy volunteers. Clin Pharmacokinet.

[14] Kanazawa S, Ohkubo T, Sugawara K (2001). The effects of grapefruit juice on the pharmacokinetics of erythromycin. Eur J Clin Pharmacol.

[15] Limited TC. Amiloride product monograph. 2014;1–18. Available from: https://pdf.hres.ca/dpd_pm/00025095.PDF

[16] Miller AK, Harrell E, Ye L, Baptiste-Brown S, Kleim J-P, Ohrt C, Duparc S, Möhrle JJ, Webster A, Stinnett S, Hughes A, Griffith S, Beelen AP (2013). Pharmacokinetic interactions and safety evaluations of coadministered tafenoquine and chloroquine in healthy subjects. Br J Clin Pharmacol.

[17] Inc WP. Pantoprazole product monograph. 2012;1–32. Available from: https://www.accessdata.fda.gov/drugsatfda_docs/label/2012/020987s045lbl.pdf

[18] Inc RL. Mycophenolate mofetil product monograph. (7):1–43. Available from: https://www.accessdata.fda.gov/drugsatfda_docs/label/2009/050722s021,050723s019,050758 s019,050759s024lbl.pdf

[19] U.S. Department of Health and Human Services FDA. In vitro drug interaction studies—cytochrome P450 enzyme- and drug interactions guidance for industry in vitro drug interaction studies—cytochrome P450 enzyme- and transporter-mediated drug interactions guidance for industry. 2020;(January). Available from: https://www.fda.gov/media/134582/download

[20] Giacomini MM, Hao J, Liang X, Chandrasekhar J, Twelves J, Whitney JA, Lepist EI, Ray AS (2017). Interaction of 2,4-diaminopyrimidine-containing drugs including fedratinib and trimethoprim with thiamine transporters. Drug Metab Dispos.

[21] Chen L, Shu Y, Liang X, Chen EC, Yee SW, Zur AA, Li S, Xu L, Keshari KR, Lin MJ, Chien HC, Zhang Y, Morrissey KM, Liu J, Ostrem J, Younger NS, Kurhanewicz J, Shokat KM, Ashrafi K, Giacomini KM (2014). OCT1 is a high-capacity thiamine transporter that regulates hepatic steatosis and is a target of metformin. Proc Natl Acad Sci U S A.

[22] Liang X, Yee SW, Chien H-C, Chen EC, Luo Q, Zou L, Piao M, Mifune A, Chen L, Calvert ME, King S, Norheim F, Abad J, Krauss RM, Giacomini KM (2018). Organic cation transporter 1 (OCT1) modulates multiple cardiometabolic traits through effects on hepatic thiamine content. PLoS Biol.

[23] Chen L, Yee SW, Giacomini KM. OCT1 in hepatic steatosis and thiamine disposition. Vol. 14, Cell cycle (Georgetown, Tex.). 2015. p. 283–4. 10.1080/15384101.2015.1006532 PMC4353231 25590948

[24] Vora B, Green EAE, Khuri N, Ballgren F, Sirota M, Giacomini KM. Drug-nutrient interactions: discovering prescription drug inhibitors of the thiamine transporter ThTR-2 (SLC19A3). Am J Clin Nutr. 2019. 10.1093/ajcn/nqz255 PMC6944527 31764942

[25] Amr K, Pawlikowska P, Aoufouchi S, Rosselli F, El-Kamah G (2019). Whole exome sequencing identifies a new mutation in the SLC19A2 gene leading to thiamine-responsive megaloblastic anemia in an Egyptian family. Mol Genet Genomic Med.

[26] Jungtrakoon P, Shirakawa J, Buranasupkajorn P, Gupta MK, De Jesus DF, Pezzolesi MG (2019). Loss-of-function mutation in thiamine transporter 1 in a family with autosomal dominant diabetes. Diabetes..

[27] Subramanian VS, Marchant JS, Said HM (2007). Targeting and intracellular trafficking of clinically relevant hTHTR1 mutations in human cell lines. Clin Sci (Lond).

[28] Labro MT (1996). Intracellular bioactivity of macrolides. Clin Microbiol Infect Off Publ Eur Soc Clin Microbiol Infect Dis.

