# Supplementary material for: The Effects of Genetic Mutations and Drugs on the Activity of the Thiamine Transporter, SLC19A2
Source: AAPS J. 2021 Mar 1;23(2):35. doi: 10.1208/s12248-021-00562-4 (PMC7921063; doi:10.1208/s12248-021-00562-4)
Supplement: Supplementary file 1 — (DOC 290 kb) [file 12248_2021_562_MOESM1_ESM.doc]

**Supplemental Figure 1** Time-dependence of thiamine uptake by SLC19A2.


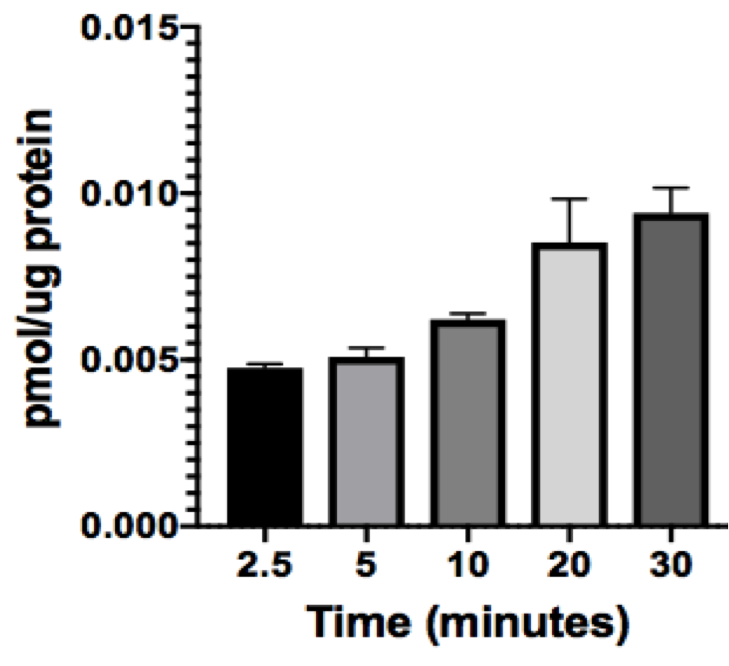


Time-dependent thiamine uptake (pmol thiamine/µg protein) in SLC19A2-overexpressing cells. Uptake of thiamine was measured at multiple time points (2.5, 5, 10, 20, and 30 min). Between 5 and 20 minutes, uptake of thiamine is linear. ug = µg

**Supplemental Figure 2** Erythromycin is a substrate of TPK1

**
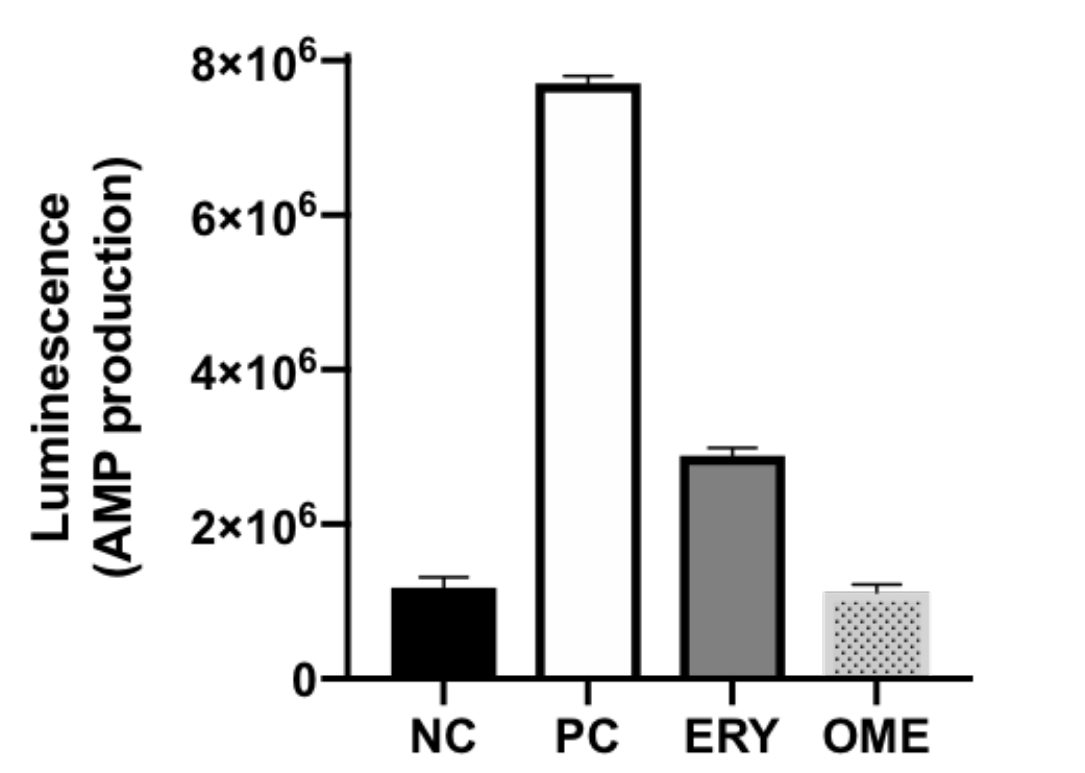
**

To determine if the top compounds in the SLC19A2 inhibitor screen (i.e., erythromycin, omeprazole) were substrates of human TPK1, we measured the level of adenosine monophosphate (AMP) generated in a TPK1 enzyme reaction using AMP-Glo™ Assay (Promega).

Luminescence was measured as a proxy for AMP production in human TPK1 reactions exposed to the following compounds: omeprazole 1mM (OME), erythromycin 1mM (ERY), thiamine 1mM (positive control or PC), and no substrate (negative control or NC). Reactions were comprised of 0.5 mL enzyme reaction mixture containing 100 μM MgCl2, 500 μM ATP, pH 7.4, 15 μg of human TPK and 1 mM of tested substrate incubated for 30 min at 37 °C. A negative control reaction was also prepared without added enzyme. Luminescence was measured with a GloMax luminometer (Promega). Luminescent signal in the erythromycin reaction was significantly higher than the negative control signal (student’s t-test, p-value < 0.0001), but still lower than the signal from the positive control. For omeprazole, the signal was not statistically different from the negative control (student’s t-test, p-value = 0.72). Figure representative of 3 independent experiments.

**Supplemental Table 1** Comprehensive list of TRMA-associated SLC19A2 missense and nonsense variants.

| TRMA-associated SLC19A2 variant* | Codon Number | Functionally characterized? | PMID |
| --- | --- | --- | --- |
| CCC-TCC/ Pro-Ser | 4 | No |  |
| GGC-CGC/ Gly-Arg | 41 | Yes | 19643445 |
| CCC-CTC/ Pro-Leu | 51 | Yes | 14994241 |
| CCC-CGC/ Pro-Arg | 60 | No |  |
| GAA-TAA/ Glu-X | 65 | No |  |
| AAT-AGT/ Asn-Ser | 71 | No |  |
| GAC-CAC/ Asp-His | 93 | Yes | 10874303 |
| CTT-TTT/ Leu-Phe | 95 | No |  |
| GGG-GAG/ Gly-Glu | 105 | Yes | 24411943 |
| AGT-GGT/ Ser-Gly | 107 | No |  |
| GAA-AAA/ Glu-Lys | 128 | Yes | 24072090 |
| GAA-AAA/ Glu-Lys | 138 | Yes | 19643445 |
| TCT-TTT/ Ser-Phe | 143 | Yes | 17463047 |
| ACA-AGA/ Thr-Arg | 158 | Yes | 16373304 |
| CGA-TGA/ Arg-X | 162 | No |  |
| AGT-TGT/ Ser-Cys | 163 | No |  |
| ACA-CCA/ Thr-Pro | 170 | No | 24355766 |
| GGC-CGC/ Gly-Arg | 172 | No | 17132746 |
| GGC-GAC/ Gly-Asp | 172 | Yes | 10391221 |
| GGT-AGT /Gly-Ser | 184 | No |  |
| GCT-GTT/ Ala-Val | 201 | Yes | 19643445 |
| ATC-TTC/ Ile-Phe | 230 | Yes | 19643445 |
| TGG-TGA/ Trp-X | 250 | No |  |
| GTG-ATG/ Val-Met | 266 | No |  |
| CGT-CTT/ Arg-Leu | 275 | No |  |
| CTT-CCT/ Leu-Pro | 282 | No |  |
| /Trp- X | 283 | No | 28371426 |
| TGG-GGG/ Trp-Gly | 320 | Yes | 24411943 |
| CGC-CTC/ Arg-Leu | 327 | No |  |
| GGT-GAT/ Gly-Asp | 334 | No | 19643445 |
| GTG-GAG/ Val-Glu | 383 | Yes | 24355766 |
| AGA-TGA/ Arg-X | 397 | Yes | 2587670 |
| ATT-ACT/ Ile-Thr | 441 | Yes | 24355766 |
| ATG-ACG/ Met-Thr | 479 | No |  |
| ACC-GCC/ Thr-Ala | 496 | No |  |

* Table only includes missense or nonsense SLC19A2 variants associated with TRMA

**Supplemental Table 2** Age and sex-matched data from patient prescribed (“on”) or not prescribed (“off”) erythromycin.

| **Erythromycin analysis** | | |
| --- | --- | --- |
|  | **On (N=4)** | **Off (N=200)** |
| **Sex** | | |
| % Female | 50 | 54 |
| % Male | 50 | 46 |
| **Age** | | |
| Mean | 32.3 | 33.8 |

The distribution of males and females and the average age in the “on” and “off” erythromycin groups is displayed. Distributions between groups are not statistically different (p-value > 0.5).

| **Supplemental Table 3** Electronic health record analysis of the most potent SLC19A2 inhibitor, erythromycin. |
| --- |
| Thiamine levels in the blood are normal in TRMA patients and SLC19A2 (-/-) knockout mice (PMID: 19879271). However, the concentration of thiamine pyrophosphate (TPP, the bioactive metabolite of thiamine) was shown to be reduced in TRMA patients compared with healthy patients (PMID: 29969779). Therefore, we used the UCSF electronic health record (EHR) database to evaluate TPP levels in the blood as a surrogate biomarker to track the effect of SLC19A2 inhibition in hematopoietic cells and tissues by clinically relevant inhibitors identified in our screen. |
| We used the UCSF Research Data Browser to search for patients with a reported TPP lab test value (measured in whole blood by the UCSF Health Clinical Laboratories). Values reported as an inequality were changed to a numerical value (i.e. < 1.6nM = 1.6nM). Lab values with missing values (i.e. DE-IDENTIFIED) and lab values without a lab collection date were excluded. |
| Patients were divided into two groups depending on their medication use. Patients prescribed erythromycin, found to be a clinically relevant inhibitor in our screen, were grouped into the “on” drug group. Search terms included “Erythromycin”, “Ery-tab”, “Eryc”, “Erythrocin”, “Eryped”, and “E.E.S.” for erythromycin. Medication orders whose route of administration was topical, ophthalmic, and miscellaneous as well as orders without a medication order start date were excluded (Figure 5A). The remaining patients (i.e. individuals never prescribed erythromycin) were grouped into the “off” drug group. To prevent counting the same patient more than once, only patients with one TPP level were included in the “off” group, which reduced our sample size to 1,278 individuals for the “off” drug group (Figure 5A). |
| Patients in the “on” drug group were further filtered based on the laboratory collection date relative to the first medication order start date. TPP levels measured within 7 to 60 days after the first medication order start date were included. A minimum of 7 days between medication start date and the TPP level was chosen to allow time for an effect to be observed because depletion of thiamine stores in the human body can take between 2 - 4 weeks. For patients with more than one lab value, only the lab value closest to the first medication order start date was included. Lastly, patients were age- and sex-matched using the MatchIt package in R to be consistent in both the “on” and “off” groups which resulted in a final sample sizes of 4 patients “on” drug and 200 patients “off” drug for the erythromycin analysis (Supplemental Table 2). |
| Welch’s two sample t-test was performed to examine significance between groups (Figure 5B); ggplot package in R (version 3.4.0) and GraphPad Prism 7 were used to plot the data. |

**Supplemental Table 4 The 63 drugs associated with drug-induced megaloblastic anemia and their respective percent SLC19A2 thiamine uptake.**

| Drug | Average % SLC19A2 thiamine uptake |
| --- | --- |
| Fedratinib | 20.96569079 |
| Omeprazole | 26.59445439 |
| Amprolium | 29.11586224 |
| Erythromycin | 29.79655745 |
| Trimethoprim | 31.70526485 |
| Mycophenolate mofetil | 32.83134592 |
| Chloroquine | 44.013215 |
| Amiloride | 44.84047242 |
| Lansoprazole | 45.86692016 |
| Pantoprazole | 46.45906647 |
| Quinidine | 53.05 |
| Rifampicin | 56.091445 |
| Leflunomide | 57.6845146 |
| Esomeprazole | 57.6999625 |
| Clarithromycin | 58.24267695 |
| Gemcitabine | 63.32164797 |
| Artemether | 67.43888736 |
| Doxycycline | 67.76320659 |
| Proguanil | 68.77720884 |
| Quinine | 69.66666667 |
| Fludarabine | 71.10952024 |
| Famotidine | 74.0302927 |
| Metformin | 74.21795244 |
| Phenytoin | 74.96618871 |
| Rabeprazole | 75.32281374 |
| Primaquine | 76.45052113 |
| Linezolid | 77.685 |
| Doxycycline | 77.78956553 |
| Azithromycin | 81.90204483 |
| Gentamicin | 81.905 |
| Phenformin | 82.3023348 |
| Mycophenolic acid | 82.67 |
| Tetracycline | 83.57885081 |
| Methotrexate | 84.88701996 |
| Neomycin | 86.37 |
| Azathioprine | 87.23447204 |
| Lumafantrine | 87.72484745 |
| 5-aminosalicylic acid | 87.72666667 |
| Raltitrexed | 88.2 |
| Mercaptopurine | 90.47455336 |
| Carbenicillin | 90.78408761 |
| Chloramphenicol | 91.52333333 |
| Aminopterin | 92.95321887 |
| Thioguanine | 93.31229335 |
| 5-fluorouracil | 93.7835647 |
| Sulfadoxine | 94.78614292 |
| Teriflunomide | 95.74 |
| Colchicine | 95.9285152 |
| Amoxicillin | 95.94844017 |
| Sodium nitroprusside | 96.36 |
| Allopurinol | 96.85675941 |
| Amikacin | 98.745 |
| Pyrimethamine | 99.18347013 |
| Nitrofuranatoin | 101.075 |
| Sulfasalazine | 101.3065363 |
| Ampicillin | 102.3276487 |
| Hydroxyurea | 105.5 |
| Cimetidine | 107.3914618 |
| Isoniazid | 107.9976315 |
| Oxacillin | 109.7376742 |
| Triamterene | 110.5058857 |
| Ranitidine | 110.5134419 |
| Estradiol | 112.178465 |
